# Analytics in Extrusion-Based Bioprinting: Standardized Methods Improving Quantification and Comparability of the Performance of Bioinks

**DOI:** 10.3390/polym15081829

**Published:** 2023-04-09

**Authors:** Svenja Strauß, David Grijalva Garces, Jürgen Hubbuch

**Affiliations:** 1 Institute of Functional Interfaces, Karlsruhe Institute of Technology, 76344 Eggenstein-Leopoldshafen, Germany; 2 Institute of Process Engineering in Life Sciences, Section IV: Biomolecular Separation Engineering, Karlsruhe Institute of Technology, 76131 Karlsruhe, Germany

**Keywords:** alginate, bioprinting, bioink, biomaterials, fibroblasts, GelMA, printability, flow cytometry

## Abstract

Three-dimensional bioprinting and especially extrusion-based printing as a most frequently employed method in this field is constantly evolving as a discipline in regenerative medicine and tissue engineering. However, the lack of relevant standardized analytics does not yet allow an easy comparison and transfer of knowledge between laboratories regarding newly developed bioinks and printing processes. This work revolves around the establishment of a standardized method, which enables the comparability of printed structures by controlling for the extrusion rate based on the specific flow behavior of each bioink. Furthermore, printing performance was evaluated by image-processing tools to verify the printing accuracy for lines, circles, and angles. In addition, and complementary to the accuracy metrics, a dead/live staining of embedded cells was performed to investigate the effect of the process on cell viability. Two bioinks, based on alginate and gelatin methacryloyl, which differed in 1% (*w*/*v*) alginate content, were tested for printing performance. The automated image processing tool reduced the analytical time while increasing reproducibility and objectivity during the identification of printed objects. During evaluation of the processing effect of the mixing of cell viability, NIH 3T3 fibroblasts were stained and analyzed after the mixing procedure and after the extrusion process using a flow cytometer, which evaluated a high number of cells. It could be observed that the small increase in alginate content made little difference in the printing accuracy but had a considerable strong effect on cell viability after both processing steps.

## 1. Introduction

Artificially generated scaffolds loaded with cellular material in the field of tissue engineering find their application as implants to replace damaged tissue and as models to study diseases or the effect of active compounds [1,2]. In this context, hydrogels are commonly employed, as these biomaterials form a highly swollen network in the aqueous phase resembling the physical structure of the extracellular matrix [3]. The biochemical composition of such tissue analog can be formulated according to the specific application. Therefore, naturally derived polymers, such as polysaccharides, e.g., alginate and hyaluronic acid, as well as proteins, e.g., collagen and gelatin, are suitable components [4]. Biofabrication methods, such as three-dimensional (3D) bioprinting, have gained attention in this field, as a wide range of material compositions can be used, and the method is relatively easy to scale-up [5]. So-called bioinks are produced for this purpose, which comprise polymer solutions with embedded cells [6]. Extrusion-based bioprinting (EBB) processes have to fulfill several requirements for the layer-by-layer generation of 3D structures intended for the application as tissue analogs. From the structural point of view, predefined geometries, which can be traced back to a series of simple structures that are stacked on top of each other, have to be produced accurately. In the final stages, the bioinks should retain the fabricated shape. During and after the printing process, high cell viability has to be maintained as the cells undergo mechanical stress during the process. The created network must support both vascularization and metabolic activities during cell culture. Meeting the structural and biological requirements has been accepted as a compromise. This is because parameters that increase the stability of printed scaffolds have a negative impact on cell viability, as those increase shear stress [2,7,8]. Although the rheological characterization of bioinks is a standard in bioprinting, the gained information is rarely exploited in the settings of printing parameters [9,10,11]. The common systematics of printability studies relies on a constant pneumatic pressure and/or printing speed while using different bioink compositions [9,12,13], and no relationship between those parameters is presented. Other studies simply set the printing parameters for each tested bioink in a rather arbitrary manner [11,14]. Furthermore, the aforementioned approaches may result in unequal amounts of extruded material since the rheological properties of the bioinks depend on the polymer content and the type of polymer. Therefore, such methodologies do not allow an adequate comparison of the structural characteristics from the printed scaffolds based solely on the material properties. This issue was addressed in the review by Gillispie et al. [15]. Furthermore, methodologies for the evaluation of printed structures are lacking standards as well. Methods in that field consist of the manual extraction of the metrics of the produced structures [16,17,18,19,20]. Such methods are prone to observer-dependent errors and are not reproducible. One possible post-printing analysis method circumventing this drawback is automated image analysis. This method shows the advantages of automation, leading to higher reproducibility as well as objectivity. In addition, images and extracted data undergo long-time storage. These methods are well established for quality control in production processes [21,22]. There have been advances in the image analysis field, but workflows still include several manual observer-dependent steps [18]. The determination of cellular viability mostly consists of evaluation of images acquired by microscopy. On the one hand, specific characteristics, such as the morphology and size of the cells can be extracted from the images, but on the other hand, the images represent only a small portion of the printed structure. Studies in the field are limited to a low number of cells in the range of hundreds of cells [23,24]. The reproducibility as well as the significance of the results regarding cell viability can be increased by the implementation of automated image processing workflows, as well [25,26]. A further option to quantify several thousand cells is flow cytometry as already tested in a few studies in the field [27,28]. The lack of standardized printing methodologies as well as techniques for the evaluation of the printing performance of bioinks presents a limitation in the development of formulations as a comparison within and between laboratories.

In this work, we aim to establish robust and objective methods for the evaluation of the printing performance of biomaterial inks and bioinks. The rheological properties of two biomaterial inks were characterized in order to determine the relationship between the printing speed and pneumatic pressure of each biomaterial ink and, therefore, control the flow rate within experiments. The assessment of the printing performance considered the accuracy of printing single-layer structures, i.e., lines, circles, and angles. For this purpose, automated image processing workflows were developed to extract geometrical features from the produced structures. The developed tools were used in order to characterize the effect of the bioink composition as well as the presence of cells on the resulting geometry. As a second part of the investigation of the printing performance, the viability of cells embedded in the bioink was determined directly after the mixing step and after bioprinting via staining and data acquisition by means of flow cytometry.

## 2. Materials and Methods

### 2.1. Cell Culture

NIH 3T3 mouse fibroblasts (CLS Cell Lines Service GmbH, Eppelheim, Germany) were incubated in Dulbecco’s Modified Eagle Medium (DMEM, high glucose, GlutaMAX™) supplemented with 10% (*w*/*v*) fetal bovine serum (FBS), 50 U mL^−1^ penicillin, and 50 µg mL^−1^ streptomycin. Cell culture media and supplements were acquired from Gibco™(Thermo Fisher Scientific, Waltham, MA, USA). Fibroblasts were cultured in tissue culture (TC) flasks at 37 ${}^{\circ }C$ in a humidified atmosphere containing 5% ${\mathrm{CO}}_{2}$. Cells were passaged upon reaching 70% to 80% confluency.

### 2.2. Biomaterial Ink and Bioink Preparation

Gelatin methacryloyl (GelMA) was produced from Type A Gelatin (300 bloom strength, Sigma-Aldrich, St. Louis, MO, USA) according to the method published by Grijalva Garces et al. [29]. The degree of functionalization of used GelMA was 65% determined by the method by Habeeb [30]. Alginic acid sodium salt was obtained from Sigma-Aldrich (A2033, from brown algae). The adequate quantities of biomaterial were dissolved in Dulbecco’s Phosphate Buffered Saline (DPBS, without calcium and magnesium, Thermo Fisher Scientific) and mixed at 3500 rpm for 5 min in a SpeedMixer^®^ (Hauschild GmbH & Co. KG, Hamm, Germany). The biomaterial ink was filled directly to a final volume of 3 mL into 10 mL cartridges (Nordson Corporation, Westlake, OH, USA) for cell-free printing. Not all formulations employed in this study contained cells, which is why they are referred to as ‘biomaterial inks’ instead of ‘bioink’ for the sake of clarity. Alginic acid and GelMA solutions for the cell-containing experiments were prepared as mentioned above. However, the initial concentration was higher, taking into account the dilution of the biomaterial ink after the mixing of the cell suspension with the cell-free biomaterial ink. The concentrated biomaterial ink was transferred to 5 mL syringes (B. Braun SE, Melsungen, Germany). Cells were harvested from the TC flasks with trypsin/ethyleneaminetetraacetic acid (Gibco™), centrifuged at 300 rcf for 5 min, and resuspended in 150 μL fresh DPBS. Subsequently, the cell suspension was transferred into 3 mL syringes (B.Braun). The syringe filled with biomaterial ink and the cell containing syringe were connected via a Luer-Lock adapter and mixed by pushing five times back and forth between both syringes. The cell-laden bioink was then loaded into the 10 mL cartridges for printing and sealed with pneumatic pistons (Nordson Corp.). The first formulation was prepared with 3% (*w*/*v*) alginate and with 3% (*w*/*v*) GelMA (A3G3), the second 4% (*w*/*v*) alginate and 3% (*w*/*v*) GelMA (A4G3). The final cell count was set to 2 × 10^6^ cells mL^−1^ for the bioinks. A summary of the formulations with the respective components is shown in Table 1.

### 2.3. Rheological Characterization

The rheological behavior of the polymer solutions was characterized based on the shear rate-dependent viscosity. For this purpose, a rotational rheometer Physica MCR301 (Anton Paar GmbH, Graz, Austria) with a cone-plate geometry (diameter 25 mm, cone angle 1${}^{\circ }$) was used. Additionally, a solvent trap was used to prevent the sample drying during measurements. The viscosity of both cell-free formulations, i.e., A3G3 and A4G3, was determined as a function of the shear rate in the range of 1 × 10^−1^ s^−1^ to 1 × 10^3^ s^−1^. The data of the viscous behavior were fitted according to the Ostwald–de Waele relationship, and the values of the power law exponent *n* and the consistency index *K* were determined for this model using Origin 2021 (OriginLab Corporation, Northampton, MA, USA). The rheological characterization was carried out as three biological replicates (n=3) with a set of three technical triplicates (n=3) each, resulting in data sets of nine replicates. For each biological replicate, an independently prepared solution was used.

In order to control the extrusion rate, i.e., the deposited material amount, the Ostwald–de Waele relationship was coupled with the Hagen–Poiseuille equation for the volumetric flow rate *Q* through a cylindrical capillary, see Equation (1), where *R* and *l* represent the capillary radius and length, respectively. The geometric variables of the capillary were taken according to the nozzle (inner diameter 0.61 mm, length 12.7 mm, Nordson Corp.) intended for the printing process. For evaluation of the printing accuracy in Section 2.4.1, the printing speed was set equal to the mean velocity $\bar{v}$ during extrusion at 10 mm s^−1^, and the required pneumatic pressure *p* was calculated. The pneumatic pressures used for printing of both formulations are given in Table 1.

(1) $$Q=\bar{v}\;\pi R^{2}=\frac{n\pi }{3n+1}{\left(\frac{R^{3n+1}\;p}{2\;K\;l}\right)}^{1/n}$$

### 2.4. Printing Performance Evaluation

A key aspect of bioprinting is the reproducibility and accurate fabrication of scaffolds while maintaining a high viability of the cells within the bioink. Therefore, the characterization of the printing performance consists of two parts, namely, the assessment of the printing accuracy, where the printed structures were compared to the predefined computer-aided design (CAD) model, and the determination of the living and dead cell counts after the extrusion process via fluorescent staining.

All printing and extrusion experiments were performed with a BioScaffolder 3.1 bioprinter (GeSiM mbH, Radeberg, Germany). The cartridge with the attached stainless-steel nozzle (inner diameter 0.61 mm, length 12.7 mm, Nordson Corp.) was placed on the holder and connected to the air supply. The structures were printed on glass microscopy slides with a distance of 0.5 mm to the nozzle tip. The applied pneumatic pressures were calculated as mentioned in Section 2.3. The specific values are listed in Table 1. The applied pressure was verified beforehand with a barometer Go Direct^®^ (Vernier Software & Technology, Beaverton, OR, USA), and adjusted, if required.

#### 2.4.1. Printing Accuracy Assessment

The aim of the printing accuracy assessment was to find out how accurately the printed structure matches the CAD model. Three single-layer structures, namely a line, a circle, and an object consisting of multiple angles, were printed, imaged, and analyzed. An overview of the objects with corresponding dimensions is given in Table 2.

**Figure 1:**
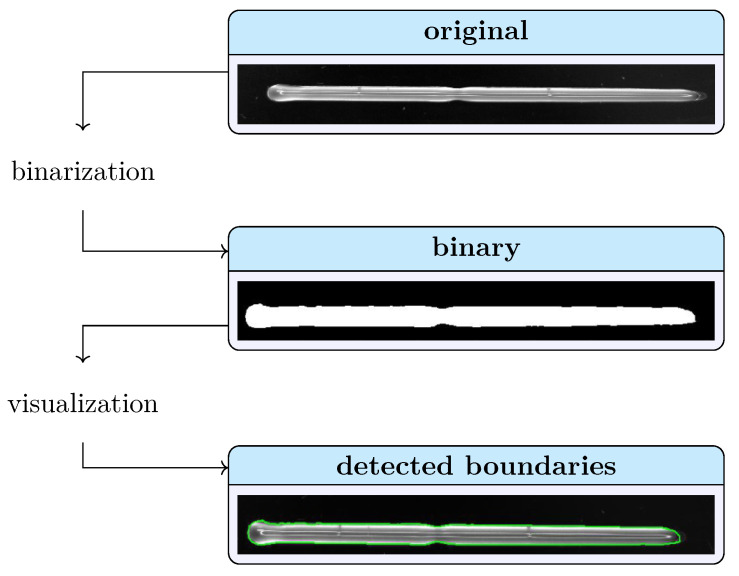
Exemplary representation of the object recognition. The original image (**top**) is binarized using local thresholding algorithms (**middle**), and an overlay with the detected boundaries of the identified structure on the original image was created as a visual control (**bottom**).

Each structure was printed as a set of 3 biological replicates (n=3) and 8 technical replicates (n=8) resulting in total in 24 objects per bioink and biomaterial ink. The biological replicates of the cell-free experiments consisted of independently prepared biomaterial inks. For the bioinks, cells were additionally harvested directly prior to the mixing step from independent TC flasks. Each object was printed on glass microscope slides. Immediately after printing, each object was individually photographed. For image acquisition, a monochrome camera (DALSA GENIE NANO-M2420, Stemmer Imaging AG, Puchheim, Germany) with 52 pixel/mm was used. The object was placed on a slide on a black background and illuminated with a white light-emitting diode (LED) ring light (CCS HPR2-150 SW, Stemmer Imaging AG). In order to obtain an objective quantification of the printed geometries, an image processing workflow was developed using Matlab^®^ R2022a (TheMathWorks Inc., Natick, MA, USA). In a first step, all images were binarized using local thresholding algorithms. Therefore, each pixel was replaced by the median value of the 3-by-3 surrounding pixels. The image processing workflow is shown schematically in Figure 1. Subsequently, the binary images were cropped to the region of interest depending on the structure. From then on, the objects were segmented, and images were evaluated for the specific structure characteristics.

The parameters extracted based on the image files are also listed in Table 2. The accuracy of printing all three geometries was evaluated by the normalized width $w_{n}$, i.e., the filament width *w* divided by the diameter of the nozzle. In the case of the line geometry, the filament width was calculated as the mean pixel count along the line. The filament width of the printed circles was measured at 180${}^{\circ }$ from the starting point and 2% of the circle was evaluated. The line width of the angle composite structure was determined at five different points along the printed structure. The analysis of the line also included the normalized length $l_{n}$. Therefore, the length of the line *l* was divided by the length of the CAD model of 30 mm. The assessment of the accuracy of the printed circles and angle composite structures was performed by including a midline as a reference and by combining the inner and outer boundaries of the structure into a single parameter. The midline represents the center of the nozzle, i.e., the axis of movement of the printer equipment. In the case of the circles, the normalized radii to midline $r_{n}$ were calculated as the sum of inner radius $r_{i}$ and outer radius $r_{a}$ detected on the binary image divided by two times the circle radius, i.e., 15 mm as designed in CAD. The 3 mm diameter circle was needed solely as a starting point for the image processing workflow. The angle composite structure was evaluated by the normalized angle $\alpha_{n}$. This parameter was calculated as the sum of each inner angle $\alpha_{i}$ and outer angle $\alpha_{o}$ of the boundaries on the binary image divided by two times the corresponding angle on the CAD file.

#### 2.4.2. Effect of Processing on Cell Viability

**Figure 2:**
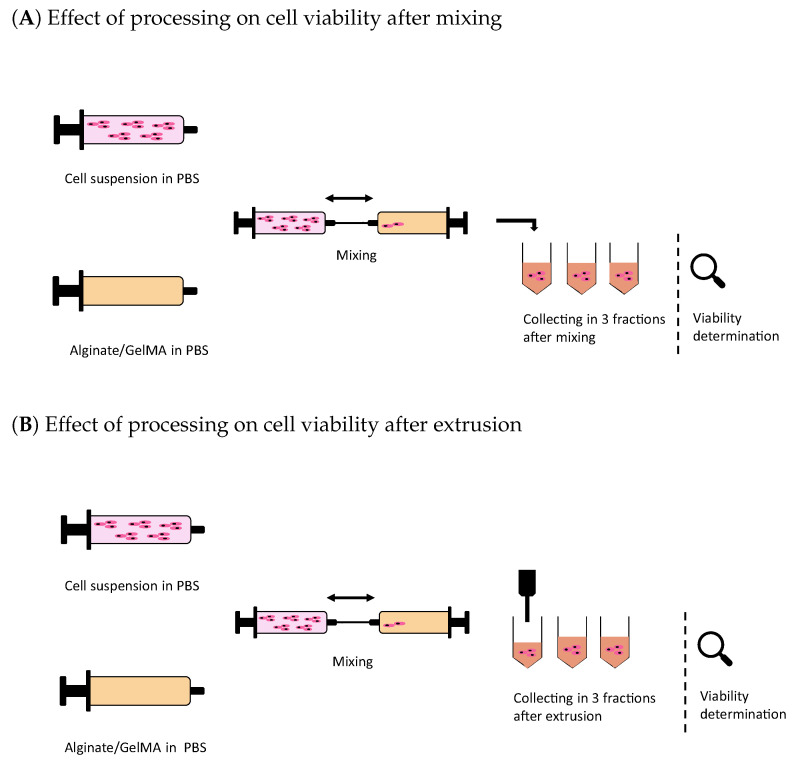
Schematic of the workflow applied for the determination of the processing effects on cell viability. A biological replicate consisted of cells harvested independently and mixed with the biomaterial ink. In (**A**), the processing ended after mixing the cellular material into the biomaterial ink, which was collected in three fractions. In (**B**), the processing of the bioink consisted of mixing it with the cell suspension and extrusion with the printer. The extruded bioink was collected in three fractions. Each collected fractions was diluted separately before cellular staining and data acquisition with a flow cytometer. Three biological replicates of each process were performed; therefore, each biological replicate was completed before starting the next replicate.

A high cell viability is essential throughout the manufacturing process of cell-loaded scaffolds. The effect of processing on the cell viability was determined after a mixing step and after extrusion. The analytical methodology for the determination of the cell viability is schematically shown in Figure 2.

The bioink was prepared as mentioned in Section 2.2. Three fractions were collected after mixing, weighed, and subsequently diluted 20-fold by weight with DPBS. Similarly, the bioink was extruded as three fractions, which were diluted with DPBS, as well. Fluorescent stains were added to the diluted bioink to a final concentration of 0.1 μM calcein-AM and 1.5 μM propidium iodide (PI); both stains were purchased from Invitrogen (Thermo Fisher Scientific). The samples were analyzed after an incubation of 15 min at room temperature. Data acquisition was performed with a MACSQuant^®^ Analyzer 10 flow cytometer (Miltenyi Biotec B.V. & Co. KG, Bergisch Gladbach, Germany) at 100 mL min^−1^. The acquired data were gated using FlowJo (Becton, Dickinson & Company, Ashland, OR, USA). This step was used to exclude debris and agglomerates. The distinction between live and dead cells was based on the green and red signals, respectively. Dead cells were gated using a control sample with fixated and permeabilized cells before staining with PI. For fixation, the cells were resuspended in a 3.7% (*v*/*v*) paraformaldehyde (AppliChem GmbH, Darmstadt, Germany) in DPBS solution and incubated for 10 min. After a wash step with DPBS, the cells were permeabilized with a 0.1% (*v*/*v*) Triton-X (Sigma-Aldrich) in DPBS solution for 15 min. Then, a further wash step was proceeded. Fixation and permeabilization were performed at room temperature. The experiment to monitor the effect of the extrusion process on the cells consisted of three biological replicates (n=3); therefore, independently prepared polymer solutions were used. Additionally, cells were harvested directly prior to the mixing step from a separate TC flask. Each fraction of diluted bioink containing stained cells (n=3) was analyzed as technical triplicates (n=3) resulting in data sets of 27 values. The processing steps and the subsequent determination of cell viability were completed before starting a new biological replicate. The cell viability was calculated as the number of viable cells divided by the total number of cells, i.e., both viable and dead cells present in a single technical replicate. The analytical methodology for the determination of the cell viability is schematically shown in Figure 2.

### 2.5. Data Handling and Statistical Analysis

Data evaluation, image processing, data visualization, and statistical analysis were performed with Matlab^®^ R2022a (TheMathWorks Inc., Natick, MA, USA). Errors related to the calculated parameters were determined after the first-order Taylor series method for uncertainty propagation. The normal distribution of data sets was verified using the Jarque–Bera test with an α-value set to 0.05. A one-way analysis of variance (ANOVA) was carried out in order to find significant differences, and a *p*-value below 0.05 was classified as statistically significant.

## 3. Results and Discussion

### 3.1. Rheological Characterization

**Figure 3:**
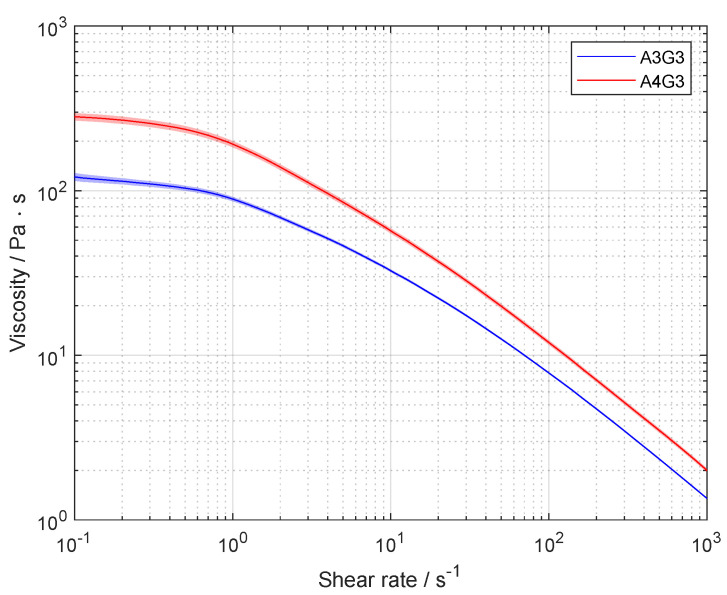
Rheological characterization of biomaterial inks A3G3 and A4G3. Viscosity is shown as a function of the shear rate. The results are presented as mean values, the shaded areas, the standard deviation. Each formulation was prepared separately for three times (n=3), and three technical replicates were tested (n=3) from each batch, resulting in data sets of nine values.

The rheological behavior of the bioink has a great influence on the process as the biomaterial flows through a nozzle and induces stress on the embedded cells. Hence, the viscosity was determined for the cell-free biomaterial inks A3G3 and A4G3 as a function of the shear rate in the range of 1 × 10^−1^ s^−1^ to 1 × 10^3^ s^−1^. Figure 3 provides the associated results.

Both biomaterial inks showed a similar non-Newtonian behavior at different magnitudes. The course of the viscosity functions showed a plateau at the low range of the shear rate and a shear thinning regime with an increasing shear rate. The viscosity plateaus had values of about 150 Pa s, and 300 Pa s for biomaterial inks A3G3 and A4G3, respectively. The shear thinning regime was fitted using the Ostwald–de Waele relationship to better describe the behavior of the biomaterial inks during extrusion. The power law exponent *n* and the consistency index *K* are listed in Table 1. The increase in viscosity of A4G3 by a factor of up to 1.9 in comparison to the viscosity of A3G3 can be explained by the higher content of alginate in solution, as a higher amount of water is bound and the entanglement of polymers increases with concentration [31]. In order to set an equal flow rate for both formulations during extrusion, the determined fitting parameters were used to calculate the required pneumatic pressure according to the Hagen–Poiseuille equation shown in Equation (1). The results are listed in Table 1. The pressure for the extrusion experiments of the A3G3 biomaterial ink was 80.5 kPa. A higher pressure with a value of 129.1 kPa was required for the extrusion of A4G3 due to the higher viscosity of the biomaterial ink.

### 3.2. Printing Performance

#### 3.2.1. Printing Accuracy Assessment

As part of the printing performance studies, printing accuracy was evaluated as the deviation from the set printing path, taking into account the 0.61 mm nozzle diameter as well. Line, circle, and angle structures were printed and characterized by the respective geometrical features. The printed structures and evaluation parameters are presented in Table 2. For all results in this section, every structure was printed as a set of 3 biological replicates (n=3) and 8 technical replicates (n=8), resulting in a total of 24 objects per biomaterial ink and bioink. The differences between evaluation parameters were examined for statistical significance. Each bar represents the mean value with the respective standard deviation for one of the four formulations.

**Figure 4:**
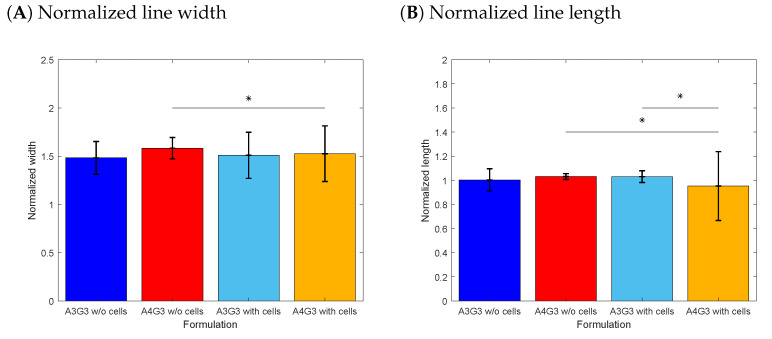
Printing accuracy study line structure. In (**A**), the normalized line width is shown, and in (**B**), the normalized line length. For all four formulations, three biological replicates (n=3) were produced, and eight technical replicates (n=8) printed for each structure, resulting in a total of 24 objects, of which the mean is shown in a bar with the associated standard deviation. Significant differences are denoted with an asterisk (p<0.05).

In Figure 4A, the normalized width in relation to the nozzle diameter for each formulation is shown. The highest normalized width was found for A4G3 without cells with a value of 1.58 ± 0.11, the lowest for A3G3 without cells with a value of 1.48 ± 0.17. Higher standard deviations are shown by the bioinks with values of 0.24 and 0.28 for the A3G3 and A4G3 formulations, respectively. The normalized width of biomaterial inks did not differ significantly. Similarly, the difference between cell-containing bioinks was not significant either. A statistical significance was observed between A3G3 without cells and A4G3 with cells. All determined values are higher than 1, as the filament thickness increases after exiting the nozzle, due to the elastic properties of polymer solutions [31]. The distances between the nozzle tip and printing surface also influence the filament width, as was shown by Habib et al. [12]. In this study, the distance was set to 0.5 mm in order for the filament to adhere to the glass surface, thus leading to thicker filaments than nozzle. In general, it is expected that the increasing viscosity of the bioink leads to thinner filaments as shown by other studies [12,17,19,32]. The increasing printing pressure is also known to increase the filament width [12,33]. In the presented study, the printing pressure was calculated according to the individual viscosity of each formulation in order to control the flow rate. The comparable normalized widths of both formulations can be attributed to the similar amount of material deposition. This approach increases the comparability of results, as printing parameters are objectively set on the basis of rheological data and not determined according to user-dependent impressions. Further methods to control the flow rate as performed by Wenger et al. [34] showed increasing reproducibility during extrusion-based bioprinting. The higher standard deviations shown by the cell-containing bioinks can be explained by air entrapment when the cells are mixed into the polymer solution between two syringes. This process is not reproducible, and air bubbles lead to inhomogeneity within the cartridge, resulting in fluctuations of the filament width. Air bubbles entrapped after the mixing steps have been addressed in the literature [15,20,26,35]. The mentioned studies implement strategies by either mixing, using a spatula or centrifuge, the cartridges or syringes to remove the entrapped air. The first imposes a non-reproducible step, while the latter can lead to redistribution or even to complete sedimentation of the cells in the bioink. For future medical applications, it is a basic requirement to develop reproducible mixing processes [20,36].

**Figure 5:**
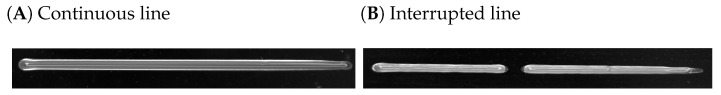
Exemplary raw images of printed lines with bioink A4G3 containing cells. In (**A**), a continuous and, in (**B**), an interrupted line is shown.

In Figure 4B, the length of the printed lines normalized to the full length of the planned structure, i.e., 3 mm, is presented for all four formulations. The highest deviation from 1 and the highest standard deviation with a value of 0.95 ± 0.29 are shown by the bioink A4G3 containing cells. The length of the line of this bioink is significantly different than that of A3G3 without cells and A3G3 with cells. The sample set of printed lines of the A4G3 bioink containing cells includes four interrupted lines, and the cell-laden A3G3 bioink includes one interrupted structure, while both cell-free formulations could be printed to the full length. Values higher than 1 can be attributed to post-flow of the formulation after releasing the air pressure. The printing of non-continuous filaments can be attributed to heterogeneities in the printing cartridge that arise during the mixing of cells by the two-syringe method as air is introduced into and entrapped in the mixture. Exemplary images of a continuous as well as an interrupted line are depicted in Figure 5.

**Figure 6:**
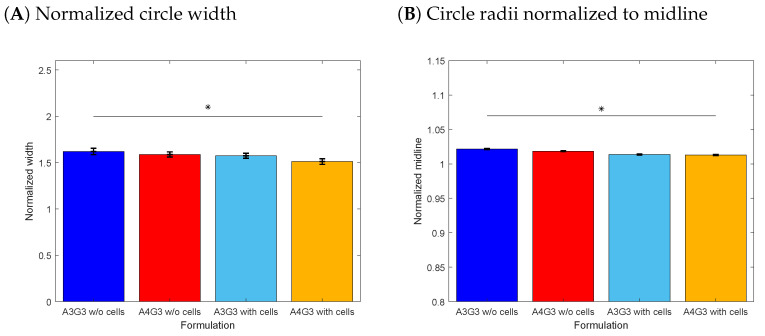
Printing accuracy study for the circle structure. In (**A**), the normalized line width is shown and, in (**B**), the normalized radius to the midline. For all four formulations, three biological replicates (n=3) were produced and eight technical replicates (n=8) printed for each structure resulting in total in 24 objects, of which the mean is shown in a bar with the associated standard deviation. Significant differences are denoted with an asterisk (p<0.05).

The width of the filament in the printed circle was also determined, and was normalized to the nozzle diameter. The results are shown in Figure 6A. The greatest deviation from 1 was measured for A3G3 without cells with a value of 1.62 ± 0.03. A4G3 containing cells showed the lowest deviation with a value of 1.51 ± 0.03. A statistically significance difference was found between both values. Comparing the normalized width of the bioinks containing cells, the values did not differ significantly. Similarly, no statistically significant differences were observed between the cell-free biomaterial inks. Figure 6B presents the normalized radii to the midline. This parameter offers the advantage of combining two parameters, namely the inner and outer radii. The midline corresponds to the printing path, i.e., the radius of the model. In the ideal case, the inner and outer radii of the filament overlap the path line +/− half nozzle diameter. Here, the comparability of the deviation is easier with one output. Similar to the normalized width of the printed circle, the largest deviation from 1 was measured for A3G3 without cells with a value of 1.02 ± 0.0008, and the lowest, for A4G3 containing cells with a value of 1.01 ± 0.0007. The radii normalized to the midline of both compositions differed significantly, as well.

The lack of differences between the normalized widths of formulations that differ in alginate content is explained by the selection of the printing pressure to set an equal flow rate for both biomaterial inks, as previously described. The standard deviations of the normalized width are smaller compared to the standard deviations of the width of the line structure. This is due to the fact that the width of the circle is measured at only a small portion of the filament, namely 2% of the circle at 180${}^{\circ }$ from the starting point and, thus, measures fewer data points than the line structure, where the width is determined in the middle third. The higher values of the A3G3 can be explained by the lower viscosity of the A3G3 biomaterial ink, as bioinks tend to keep flowing after printing, especially as no crosslinking was performed after printing. All values are higher than 1 due to the elasticity of the polymer solution [31].

**Figure 7:**
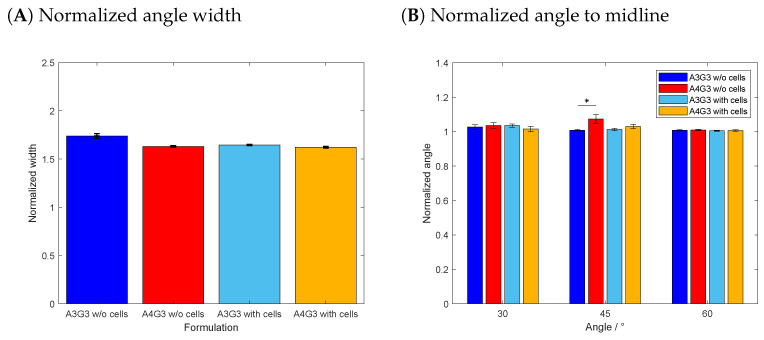
Printing accuracy study for the angle structure. In (**A**), the normalized line width is shown and, in (**B**), the normalized angle to the midline. For all four formulations, three biological replicates (n=3) were produced, and eight technical replicates (n=8) printed for each structure, resulting in a total of 24 objects, of which the mean is shown in a bar with the associated standard deviation. Significant differences are denoted with an asterisk (p<0.05).

As a final part of this study, the accuracy of printed angles was characterized by means of the normalized filament width and the normalized angle to the midline. The corresponding data are shown in Figure 7A, B, respectively. The printed structure consisted of a continuous structure with angles of 30${}^{\circ }$, 45${}^{\circ }$, and 60${}^{\circ }$. Considering the normalized filament width, the highest deviation from 1 was observed for the A3G3 without cells with a value of 1.74 ± 0.02. The lowest normalized width was produced with A4G3 containing cells with a value of 1.62 ± 0.01. No statistically significant differences between the normalized filament widths were proven between any of the tested formulations. As previously explained, there are no differences in printing accuracy due to the normalization of the volumetric flow rate.

Comparing the normalized width of the filament determined in the angle structure to the normalized width of the simple line structure, it is noteworthy that the standard deviation of the first is lower than that of the latter. The reason lies in the analytical method itself since the width of the filament of the angle structure was measured at five points along the whole structure. This data set is smaller compared to the data set used to determine the filament width along the line structure at every pixel of the evaluated section of the image. The quantification along the angle structure is less accurate, but the output serves as a rough estimate to compare whether the more complex movement of the print head has an effect on the produced structure. During the angle analysis shown in (B), the inner and outer angles were again combined together to one parameter; hence, the angles were normalized to the angle of the trajectory of the print head. The cell-free A4G3 showed the highest deviation from 1 with a value of 1.07 ± 0.03 overall. There were no significant differences between the normalized angles of all four formulations printed as a 30${}^{\circ }$ angle. Similarly, no effect of the formulation on the normalized angle was observed while printing 60${}^{\circ }$ angles. By comparing the 45${}^{\circ }$ angle, the values of the biomaterial inks A3G3 and A4G3, both without cells, differed significantly. The differences can be explained by the different alginate concentrations, as the bioinks tend to keep flowing after printing. The analysis of the printed angles was performed with an automated image processing workflow. There have been similar studies regarding printing and characterizing angular structures by He et al. [37] and Naghieh et al. [38]. Both studies state the importance of characterizing such angular patterns, as these simple patterns build up the bases of printed structures of higher complexity. However, the studies fail to give precise information on the estimation of the angles, which is a basic requirement for the standardization of processes.

It must be noted that the air pressure acting on the cartridge and the set pressure of the printing software did not match. The air pressure was, therefore, controlled with a pneumatic sensor before printing processes were started. Especially when it comes to medical applications, it is a basic requirement for the construction of 3D bioprinters that they work reliably and that the air supply is stable and not affected by the position of the printhead. There is still great potential on the side of the bioprinter manufacturers that needs to be improved. The single-layer structures characterized in this study represent the starting point since later complex geometries can be broken down into them. Characterizing the first layer is important, as defects in the base layer can prevent all subsequent layers from adhering to it. Similarly, defects on the layer can lead to the collapsing of the structure, especially with an increasing amount on layers piling on top of the basis. Future studies should include the characterization of the effect of the amount of extruded material in top of the base layer. Additionally, the strategies should be developed for the evaluation of 3D structures using automated image processing. At the moment, image acquisition takes place externally and not within the bioprinting system online. It would be desirable in the future to integrate the camera into the bioprinter with a suitable and adaptable illumination setup, as the transparency of bioinks is an issue during image acquisition that is particularly important in the choice of lighting and background to reach enough contrast. Regarding image processing, local thresholding should be preferred so that as little information as possible is lost. Such an image processing step is of particular interest if even illumination cannot be performed. Even though there were slight limitations on the imaging methods, the field of bioprinting can benefit from the application of automated image processing and analysis. The presented method proved to be robust for printed object evaluation, as it saves analytical time and reduces observer-dependent errors.

The aim of bioprinting is the production of scaffolds with high accuracy. Simultaneously, the printing of bioinks should meet the requirement of maintaining high cell viability after this process, as shear stress can induce irreversible cell damage.

#### 3.2.2. Effect of Processing on Cell Viability

**Figure 8:**
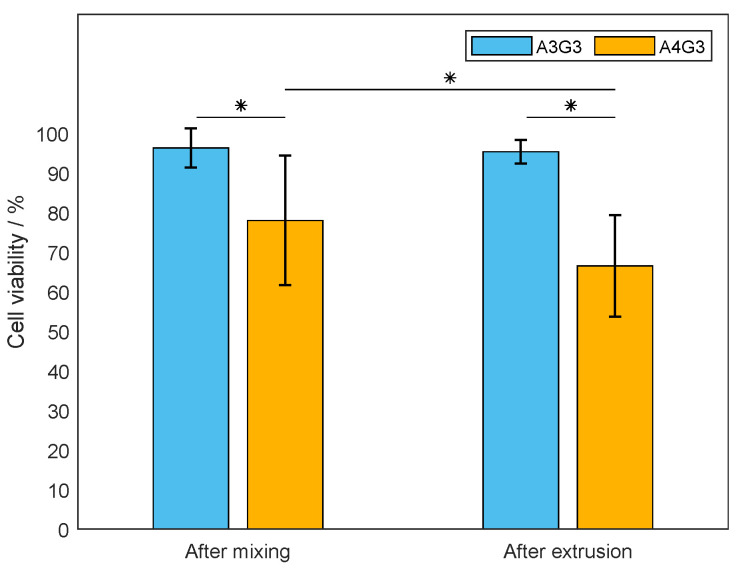
Characterization of the effect of processing on cells. The viability was determined after the mixing step and after the extrusion process. For both bioinks, the mean viability is shown as a bar with the associated standard deviation. The experiments were performed as biological triplicates (n=3). For both bioinks, each biological replicate was divided into three fractions, diluted separately (n=3), and analyzed as technical triplicates (n=3), resulting in data sets consisting of 27 values. Significant differences are denoted with an asterisk (p<0.05).

Besides the printing accuracy, the effect of processing on cell viability after the mixing process and after the extrusion process was further studied. Here, three biological replicates for A3G3 with cells and A4G3 with cells were produced and analyzed. Each cartridge containing bioink was collected in three fractions. Each fraction was diluted with DPBS, and cells were stained in the resulting suspension. For the detection of fluorescent signals and, thus, the determination of cell viability, flow cytometry was used. Each diluted fraction was analyzed in technical triplicate. The respective results are presented in Figure 8. The bar chart shows the cell viability for both tested bioinks directly after the mixing step and after the extrusion. process.

The increasing alginate content of the bioinks led to a decreasing cell viability. This effect was observed after the mixing step as well as after mixing and subsequent extrusion. The viability of cells mixed into the A3G3 formulation and into the A4G3 formulation showed a value of 96.3 ± 4.9%, and 77.9 ± 16.4%, respectively. Both values differed significantly. The viability of cells extruded within the A3G3 bioink was slightly reduced to 95.3 ± 3.0%. However, the difference was not statistically significant. Considering cells in the A4G3 formulation, viability decreased in a significant manner to 66.4 ± 12.7%. After mixing and extrusion, the viability of cells contained in the A3G3 formulation differed significantly from that of cells present in the A4G3 bioink. As cells were harvested directly prior to both processes and the proceeding analysis, the contact between cells and biomaterial was the same for each sample, i.e., effects on cell viability arose from the processing steps. The decreasing amount of viable cells can be explained by the shear stress during mixing and extrusion that leads to cell disruption. The shear stress increases with the viscosity of the polymer solution as well as with increasing pneumatic pressure. The effect of the alginate concentration and thus the increasing viscosity on decreasing cell viability is in accordance with similar studies [13,24,39]. Increasing printing pressure leading to lower cell viability was also demonstrated in further studies [13,24,40,41]. Moreover, the use of flow cytometry for data acquisition increased the precision of the determined cell viability as this value was calculated with larger amounts of counted cells. Other studies in the field of bioprinting and tissue engineering analyze a low number of microscopy images containing a low number of cells [19,23,24]. Commercially available assays, such as live/dead staining kits, lactate dehydrogenase assay, and alamar blue staining, are commonly applied in order to evaluate cell viability [42]. The assays are developed for culture methods where cells adhere to planar surfaces, i.e., two-dimensional cell culture, or cells are suspended in cell culture media. The assay components are added to the fluid phase and the molecules diffuse easily to the cells, and the absorbance or fluorescence are measured in plate readers. In tissue engineering and biofabrication, cells are embedded within a polymeric network, and the diffusion of solutes within the structures is limited. This is an issue to be considered in the application of commercially available kits. The protocols can be adapted to 3D cell culture substrates by adjusting incubation times or implementing the permeabilization of the hydrogels. By dilution of the bioink to a low viscosity cell suspension, the fast diffusion of the used stains is enabled as well as the use of the flow cytometer. Each technical replicate of this study part of the study regarding the assessment of the processing effects on viability consists of cell numbers in the range of 450 up to about 10,000. Considering the 20-fold dilution required for analysis with flow cytometry, the cell count in the bioink is calculated to be in the range between 9 × 10^3^ cells mL^−1^ and 0.2 × 10^6^ cells mL^−1^, considerably lower than the cell count of 2 × 10^6^ cells mL^−1^ to be set in the bioink. The amount of cells in each fraction differed due to the inhomogeneous mixing of the used method, where a syringe containing the cell suspension is connected to a second syringe containing the polymer solution, and a mixing effect is produced by transferring the solution back and forth between both syringes. Even though this method requires significant improvement, it was applied in the presented study, as it is commonly used in studies [43,44], and it shows the benefits of low biomaterial loss compared to static mixer components. Additionally, mixing of cells in this manner can be compliant to good manufacturing practice (GMP) conditions compared to the mixing of cells in open containers. The reduction in waste of biomaterial by improvement of the static mixing units was studied by Dani et al. [20] and should be implemented in further studies. The quantified number of cells did differ in each technical sample due to inhomogeneous mixing; however, the viability was in a similar range, as it is the ratio between viable and dead cells. As mentioned in Section 3.2.1, bioprinting can benefit from standardization of the mixing method as air bubbles are introduced in the bioink [15,20]. A further benefit is the comparability of data regarding cell viability, as the mixing method proved to be a critical step. The use of flow cytometry can also reduce observer-dependent errors while increasing reproducibility, as image acquisition mostly relies on the manual focusing of the samples during image acquisition. It is noteworthy that the use of flow cytometry is a destructive method, as the bioink has to be diluted in order to be analyzed. Microscopy is still required as a supplementary cell analytical method regarding cell adhesion, morphology, and migration during longer periods of cultivation.

Gillespie et al. [15] mentioned the limitation of the lack of control of the extruded amount of biomaterial. Measured characteristics of printed structures could differ either due to the mechanical properties of the used bioinks, or due to different amounts of bioinks deposited during printing. This presented method overcomes the mentioned difficulty by controlling the volumetric flow rate according to the specific flow behavior of each bioink. Additionally, the printing speed was set equal to the mean velocity of the biomaterial ink based on the rheological properties; thus, this parameter is directly related to the pneumatic pressure. Furthermore, the alginate content of both bioinks differed by 1% (*w*/*v*) and, thus, showed different values of viscosity. By controlling the flow rate, no differences were observed regarding the printing accuracy between both bioinks. In contrast, the increasing viscosity led to a significant decrease in cell viability directly after mixing and after extrusion. Bioinks should enable the accurate production of scaffolds while maintaining high viability and supporting biological needs. Meeting both requirements is accepted as a limitation, as increasing the concentration and thus viscosity leads to higher structural stability but increases the resulting cell disruption [2,7,32]. In this study, the bioink with the lower alginate content showed better printing performance overall, as the method compared the samples printed with a controlled flow rate. The presented methods show further advantages. The setting of printing parameters did not include the screening of printability by an observer, which is still commonly used in the bioprinting field. There are many printing parameters that can be set during a study on printing performance, and each affects the accuracy of the product in a different magnitude. Therefore, the setting of printing speed in relation to the pneumatic pressure increases the objectivity of the study, as only one was chosen arbitrarily and not both of them. The use of automated image processing to characterize printed structures reduces observer-dependent errors while saving the time required for image analysis. The presented methods increase the comparability of data between bioinks and can allow the transfer of gained knowledge between laboratories.

## 4. Conclusions

Universal methodologies should be developed and applied starting from the bioink preparation, including process analytical technology (PAT) strategies as well as standardized analytical methodologies. In this study, the focus lies on the development of analytical methods that enable the comparability of bioprinting processes regarding the metrics of printed structures as well as the effect on cell viability. First, the used bioinks and biomaterial inks were printed with the same flow rate, thus enabling the comparison of structures with the same amount of extruded material. The required pneumatic pressure was determined and set according to the specific flow behavior of each biomaterial ink that differed in alginate concentration by 1% (*w*/*v*) with a constant GelMA concentration. Additionally, the printing speed was directly related the pneumatic pressure applied during printing. This setting of printing parameters enabled the adequate comparison of printed structures produced with an equal amount of material. Second, image-processing tools were developed in order to accurately characterize the printed structures in an automated manner. The application of automated image analysis allowed the time-saving assessment of printed geometries, while reducing observer-dependent variations, and therefore, the robustness of the analytical method was enhanced. The study focused on the analysis of the first layer, as this is the base of any multi-layered structure. In the future, the developed image processing should be expanded and implemented into the characterization of 3D structures. Using the developed tools, no effect of the increasing viscosity on the structural features was shown. However, the cell mixing process did have an effect on the geometrical characteristics of the printed structures, as air was entrapped while mixing the biomaterial ink with the cellular material. Future research should include testing the control of flow rate in 3D objects, as, here, only single-layer structures were investigated. Third, cell viability was determined by flow cytometry, thereby increasing the amount of quantified cells and enhancing the precision of the acquired data compared to the conventionally used microscopy. Moreover, the increasing alginate content showed a significantly negative impact on viability during both processing steps, mixing as well as extrusion. Some issues still have to be studied, e.g., the lack of standardized and effective cell mixing processes. The used mixing strategy was the transfer of biomaterial ink and cells back and forth between two syringes, which is commonly used in the field of bioprinting. This method proved to produce an inhomogeneous cell distribution and air bubbles entrapment in the cartridge. The proposed methods show great potential for saving time and costs by eliminating the need for user-dependent print parameter screenings and enables an easy transfer between devices and laboratories. The lack of standardized methods will constitute an issue in bioprinting by the stage prior to clinical applications when meeting with regulatory agencies. The requirements include robust production processes that lead to quality attributes, independent of the location and operator.

## Figures and Tables

**Table 1:** Biomaterial ink compositions as employed in this study and abbreviations used throughout this work. Power law exponent and consistency index according to the Ostwald–de Waele relationship were determined as mentioned in Section 2.3 and used for the calculation of the specific pneumatic pressure for printing performance experiments.

Abbr.	Alginate	GelMA	Power Law	Consistency	Pneumatic Pressure
	(% (*w*/*v*))	(% (*w*/*v*))	Exponent *n*	Index *K* (Pa s^n^)	(kPa)
A3G3	3	3	0.35	146.39	80.5
A4G3	4	3	0.32	284.09	129.1

**Table 2:** Graphic models of the printing path. Images of the printed objects are taken and analyzed as a part of the printing accuracy assessment. The dimensions in the line and circle sketches are given in millimeter (mm), the dimensions in the angle sketch in degrees (${}^{\circ }$). Each structure was examined for characteristic parameters, which are shown with the respective formulae for calculation.

Structure	Evaluation Parameter	Formula/Equation	
Line	normalized width	$w_{n}=\frac{w}{d_{nozzle}}$	(2)
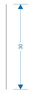	normalized length	$l_{n}=\frac{l}{l_{model}}$	(3)
Circle	normalized width	see Equation (2)	
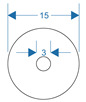	normalized radii to midline	$r_{n}=\frac{r_{i}+r_{o}}{2r_{model}}$	(4)
Angle	normalized width	see Equation (2)	
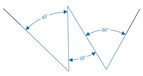	normalized angle to midline	$\alpha_{n}=\frac{\alpha_{i}+\alpha_{o}}{2\alpha_{model}}$	(5)

## Data Availability

The raw data supporting the conclusions of this article will be made available by the authors without undue reservation.

## Abbreviations

3D	Three-dimensional
A	Alginate concentration
CAD	Computer-aided design
DPBS	Dulbecco’s Phosphate Buffered Saline
EBB	Extrusion-based bioprinting
G	GelMA concentration
GelMA	Gelatin methacryloyl
GMP	Good manufacturing practice
PAT	Process analytical technology
PI	Propidium iodide
TC	Tissue culture

## References

[1] Murphy S.V., Atala A. (2014). 3D bioprinting of tissues and organs. Nat. Biotechnol..

[2] Ozbolat I.T., Hospodiuk M. (2016). Current advances and future perspectives in extrusion-based bioprinting. Biomaterials.

[3] Slaughter B.V., Khurshid S.S., Fisher O.Z., Khademhosseini A., Peppas N.A. (2009). Hydrogels in Regenerative Medicine. Adv. Mater..

[4] Thiele J., Ma Y., Bruekers S.M.C., Ma S., Huck W.T.S. (2014). 25th Anniversary Article: Designer Hydrogels for Cell Cultures: A Materials Selection Guide. Adv. Mater..

[5] Malda J., Visser J., Melchels F.P., Jüngst T., Hennink W.E., A Dhert W.J., Groll J., Hutmacher D.W. (2013). 25th Anniversary Article: Engineering Hydrogels for Biofabrication. Adv. Mater..

[6] Groll J., Burdick J.A., Cho D.W., Derby B., Gelinsky M., Heilshorn S.C., Jüngst T., Malda J., Mironov V.A., Nakayama K. (2019). A definition of bioinks and their distinction from biomaterial inks. Biofabrication.

[7] Zhao Y., Li Y., Mao S., Sun W., Yao R. (2015). The influence of printing parameters on cell survival rate and printability in microextrusion-based 3D cell printing technology. Biofabrication.

[8] Theus A.S., Ning L., Hwang B., Gil C., Chen S., Wombwell A., Mehta R., Serpooshan V. (2020). Bioprintability: Physiomechanical and Biological Requirements of Materials for 3D Bioprinting Processes. Polymers.

[9] Liu W., Heinrich M.A., Zhou Y., Akpek A., Hu N., Liu X., Guan X., Zhong Z., Jin X., Khademhosseini A. (2017). Extrusion Bioprinting of Shear-Thinning Gelatin Methacryloyl Bioinks. Adv. Healthc. Mater..

[10] Erdem A., Darabi M.A., Nasiri R., Sangabathuni S., Ertas Y.N., Alem H., Hosseini V., Shamloo A., Nasr A.S., Ahadian S. (2020). 3D Bioprinting of Oxygenated Cell-Laden Gelatin Methacryloyl Constructs. Adv. Healthc. Mater..

[11] Jongprasitkul H., Turunen S., Parihar V.S., Kellomäki M. (2022). Two-step crosslinking to enhance the printability of methacrylated gellan gum biomaterial ink for extrusion-based 3D bioprinting. Bioprinting.

[12] Habib A., Sathish V., Mallik S., Khoda B. (2018). 3D printability of alginate-carboxymethyl cellulose hydrogel. Materials.

[13] Mondal A., Gebeyehu A., Miranda M., Bahadur D., Patel N., Ramakrishnan S., Rishi A.K., Singh M. (2019). Characterization and printability of Sodium alginate-Gelatin hydrogel for bioprinting NSCLC co-culture. Sci. Rep..

[14] Lee B.H., Lum N., Seow L.Y., Lim P.Q., Tan L.P. (2016). Synthesis and characterization of types A and B gelatin methacryloyl for bioink applications. Materials.

[15] Gillispie G., Prim P., Copus J., Fisher J., Mikos A.G., Yoo J.J., Atala A., Lee S.J. (2020). Assessment methodologies for extrusion-based bioink printability. Biofabrication.

[16] Gao T., Gillispie G.J., Copus J.S., Kumar A.P., Seol Y.J., Atala A., Yoo J.J., Lee S.J. (2018). Optimization of gelatin-alginate composite bioink printability using rheological parameters: A systematic approach. Biofabrication.

[17] Giuseppe M.D., Law N., Webb B., Macrae R.A., Liew L.J., Sercombe T.B., Dilley R.J., Doyle B.J. (2018). Mechanical behaviour of alginate-gelatin hydrogels for 3D bioprinting. J. Mech. Behav. Biomed. Mater..

[18] Uzun-Per M., Gillispie G.J., Tavolara T.E., Yoo J.J., Atala A., Gurcan M.N., Lee S.J., Niazi M.K.K. (2020). Automated Image Analysis Methodologies to Compute Bioink Printability. Adv. Eng. Mater..

[19] Aldana A.A., Valente F., Dilley R., Doyle B. (2021). Development of 3D bioprinted GelMA-alginate hydrogels with tunable mechanical properties. Bioprinting.

[20] Dani S., Ahlfeld T., Albrecht F., Duin S., Kluger P., Lode A., Gelinsky M. (2021). Homogeneous and reproducible mixing of highly viscous biomaterial inks and cell suspensions to create bioinks. Gels.

[21] Malamas E.N., Petrakis E.G., Zervakis M., Petit L., Legat J.D. (2003). A survey on industrial vision systems, applications and tools. Image Vis. Comput..

[22] Semeniuta O., Dransfeld S., Martinsen K., Falkman P. (2018). Towards increased intelligence and automatic improvement in industrial vision systems. Procedia CIRP.

[23] Schuurman W., Levett P.A., Pot M.W., van Weeren P.R., Dhert W.J.A., Hutmacher D.W., Melchels F.P.W., Klein T.J., Malda J. (2013). Gelatin-Methacrylamide Hydrogels as Potential Biomaterials for Fabrication of Tissue-Engineered Cartilage Constructs. Macromol. Biosci..

[24] Koch F., Tröndle K., Finkenzeller G., Zengerle R., Zimmermann S., Koltay P. (2020). Generic method of printing window adjustment for extrusion-based 3D-bioprinting to maintain high viability of mesenchymal stem cells in an alginate-gelatin hydrogel. Bioprinting.

[25] Di Z., Klop M.J.D., Rogkoti V.M., Le Dévédec S.E., van de Water B., Verbeek F.J., Price L.S., Meerman J.H.N. (2014). Ultra High Content Image Analysis and Phenotype Profiling of 3D Cultured Micro-Tissues. PLoS ONE.

[26] Eggert S., Hutmacher D.W. (2019). In vitro disease models 4.0 via automation and high-throughput processing. Biofabrication.

[27] Gretzinger S., Beckert N., Gleadall A., Lee-Thedieck C., Hubbuch J. (2018). 3D bioprinting—Flow cytometry as analytical strategy for 3D cell structures. Bioprinting.

[28] Godar D.E. (2018). 3D Bioprinting: Surviving under Pressure. Tissue Regen..

[29] Grijalva Garces D., Radtke C.P., Hubbuch J. (2022). A Novel Approach for the Manufacturing of Gelatin-Methacryloyl. Polymers.

[30] Habeeb A.F. (1966). Determination of free amino groups in proteins by trinitrobenzenesulfonic acid. Anal. Biochem..

[31] Münstedt H., Schwarzl F.R. (2014). Deformation and Flow of Polymeric Materials.

[32] Chung J.H., Naficy S., Yue Z., Kapsa R., Quigley A., Moulton S.E., Wallace G.G. (2013). Bio-ink properties and printability for extrusion printing living cells. Biomater. Sci..

[33] Webb B., Doyle B.J. (2017). Parameter optimization for 3D bioprinting of hydrogels. Bioprinting.

[34] Wenger L., Strauß S., Hubbuch J. (2022). Bioprinting Automated and dynamic extrusion pressure adjustment based on real-time flow rate measurements for precise ink dispensing in 3D bioprinting. Bioprinting.

[35] Paxton N., Smolan W., Böck T., Melchels F., Groll J., Jungst T. (2017). Proposal to assess printability of bioinks for extrusion-based bioprinting and evaluation of rheological properties governing bioprintability. Biofabrication.

[36] Cohen D.L., Lo W., Tsavaris A., Peng D., Lipson H., Bonassar L.J. (2011). Increased Mixing Improves Hydrogel Homogeneity and Quality of Three-Dimensional Printed Constructs. Tissue Eng. Part C Methods.

[37] He Y., Yang F., Zhao H., Gao Q., Xia B., Fu J. (2016). Research on the printability of hydrogels in 3D bioprinting. Sci. Rep..

[38] Naghieh S., Sarker M., Sharma N.K., Barhoumi Z., Chen X. (2019). Printability of 3D Printed Hydrogel Scaffolds: Influence of Hydrogel Composition and Printing Parameters. Appl. Sci..

[39] Tabriz A.G., Hermida M.A., Leslie N.R., Shu W. (2015). Three-dimensional bioprinting of complex cell laden alginate hydrogel structures. Biofabrication.

[40] Nair K., Gandhi M., Khalil S., Yan K.C., Marcolongo M., Barbee K., Sun W. (2009). Characterization of cell viability during bioprinting processes. Biotechnol. J..

[41] Snyder J., Rin Son A., Hamid Q., Wang C., Lui Y., Sun W. (2015). Mesenchymal stem cell printing and process regulated cell properties. Biofabrication.

[42] Marzi J., Fuhrmann E., Brauchle E., Singer V., Pfannstiel J., Schmidt I., Hartmann H. (2022). Non-Invasive Three-Dimensional Cell Analysis in Bioinks by Raman Imaging. ACS Appl. Mater. Interfaces.

[43] Park H., Kang S.W., Kim B.S., Mooney D.J., Lee K.Y. (2009). Shear-reversibly Crosslinked Alginate Hydrogels for Tissue Engineering. Macromol. Biosci..

[44] Hafezi F., Shorter S., Tabriz A.G., Hurt A., Elmes V., Boateng J., Douroumis D. (2020). Bioprinting and preliminary testing of highly reproducible novel bioink for potential skin regeneration. Pharmaceutics.

